# A retrospective chart review of drug treatment patterns and clinical outcomes among patients with metastatic or recurrent soft tissue sarcoma refractory to one or more prior chemotherapy treatments

**DOI:** 10.1186/s12885-015-1182-4

**Published:** 2015-03-25

**Authors:** Michael J Wagner, Leo Ismaila Amodu, Mei Sheng Duh, Caroline Korves, Franco Solleza, Stephanie C Manson, José Diaz, Maureen P Neary, George D Demetri

**Affiliations:** 1 Dana-Farber Cancer Institute, Ludwig Center at Harvard, 450 Brookline Avenue, Boston, MA 02215 USA; 2 Analysis Group, Inc, Boston, MA 02199 USA; 3 Value Evidence and Outcomes, GlaxoSmithKline, Uxbridge, UB11 1BT UK; 4 Value Evidence and Outcomes, GlaxoSmithKline, Collegeville, PA 19426 USA; 5 MD Anderson Cancer Center, Houston, TX 77030 USA; 6 North Shore-LIJ Health System, Manhasset, NY 10030 USA; 7 Novartis Pharmaceutical Corporation, East Hanover, NJ 07936 USA; 8 Novartis Pharmaceuticals, Uxbridge, UB11 1BT UK

**Keywords:** Anthracycline, Chemotherapy, Doxorubicin, Gemcitabine, Metastatic, Soft tissue sarcoma

## Abstract

**Background:**

Limited clinical data on real-world practice patterns are available for patients with metastatic/relapsed soft tissue sarcomas (STS). The primary objective of this study was to evaluate treatment patterns in patients with metastatic/relapsed STS following failure of prior chemotherapy by examining data collected from 2000 to 2011 from a major tertiary academic cancer center in the United States.

**Methods:**

Medical records, including community-based referral records, from a tertiary cancer center for adult patients with metastatic/relapsed STS with confirmed disease progression who commenced second-line treatment between January 1, 2000 and February 4, 2011, and with at least 3 months of follow-up data following second-line treatment initiation, were retrospectively reviewed. Overall survival, time to progression, and clinician-reported tumor response were collected.

**Results:**

A total of 99 patients (leiomyosarcoma, n = 48; synovial cell sarcoma, n = 7; liposarcoma, n = 5; or other histological subtypes, n = 39) received an average of four lines of treatment (maximum of 10). No consistent or dominant regimens were used in each treatment line beyond the second line. Median second-line treatment duration was 4.1 months (95% confidence interval, 3.0–5.0). Overall, 72 of 99 patients (73%) discontinued second-line treatment due to progressive disease. Median progression-free survival from initiation of second-line treatment varied across regimens from 2.0 to 6.6 months (overall median, 5.4 months).

**Conclusions:**

Wide variations in treatment were evident, with no single standard of care for patients with metastatic/relapsed STS. Most patients discontinued second-line treatment due to progressive disease, often receiving additional systemic therapy with other drugs. These data suggest a high unmet need for more efficacious treatment options and improved data collection to guide practice among patients with relapsed/refractory STS.

## Background

Soft tissue sarcomas (STS) are rare cancers of mesenchymal cell origin that include more than 50 histological subtypes, as well as many more molecularly distinct entities [1,2]. STS can resemble the differentiation of various connective tissues, including muscle fat, nerves, vessels, stromal tissues, or bone. Gastrointestinal stromal tumor (GIST) is the most common subtype of all sarcomas [3]. Other STS categories include leiomyosarcomas, liposarcomas, and pleomorphic undifferentiated sarcoma (formerly malignant fibrous histiocytoma) [3,4]. The American Cancer Society estimated that 11 280 new cases of STS were diagnosed and 3900 patients died in 2012 in the United States [4]. Survival estimates for primary localized STS depend on many factors, including anatomic location and tumor grade [1]. Despite treatment, approximately 50% of patients with STS will ultimately develop recurrences or metastatic disease [5,6].

For patients with primary resectable STS, surgery is the mainstay of treatment [7,8]. However, for patients with metastatic STS, systemic therapy with conventional cytotoxic chemotherapy remains the main treatment modality. The National Comprehensive Cancer Network [7] and the European Society for Medical Oncology [8] recommend anthracyclines (alone or combined with other agents) in most cases as first-line treatment for metastatic STS, although first-line treatment recommendations may vary by histological subtype and previous treatment. Until recently, doxorubicin was the only agent formally approved by regulatory authorities for most types of metastatic/relapsed STS [9]. Pazopanib, a multitargeted tyrosine kinase inhibitor, is now also approved for use in patients following disease progression despite prior chemotherapy based on clinical studies documenting the benefit of disease control in this population [10,11]. Several other systemic agents, including ifosfamide, gemcitabine, dacarbazine, and trabectedin, are commonly used to treat metastatic/relapsed STS, but evidence supporting these therapies is largely limited to phase II trials [12-15].

Considering the wide range of STS histological subtypes, it is difficult to draw conclusions for specific entities based on generally uncontrolled trials of heterogeneous unselected patients with STS with a variety of different treatments. In addition, information in the published literature about treatment patterns and outcomes in STS is sparse. Studies in the United States [16] and internationally [6] showed a wide variety of systemic therapy administered in patients following failure of first-line chemotherapy.

Currently, there is no globally accepted standard based on high-quality evidence for patients with advanced STS following failure of prior chemotherapy to control advanced disease. The primary objective of this study was to evaluate treatment patterns in patients with metastatic/relapsed STS following failure of prior chemotherapy by examining data collected from 2000 to 2011 from a large tertiary academic cancer center in the United States. For the purpose of this study, STS will refer to sarcomas other than GIST. As a secondary objective, this study sought to gain a high-level assessment of the clinical effectiveness of various treatments given to patients with metastatic/relapsed STS.

## Methods

This study was a retrospective analysis of patient medical records from the Dana-Farber Cancer Institute affiliated with Harvard Medical School in Boston, Massachusetts, USA, and was approved by the Dana-Farber Cancer Institute ethics committee.

All patients provided informed consent to approve the use of their medical records based on an institutional review board-approved protocol. Individual patient records were retrospectively reviewed in a sequential manner from this prospectively collected Sarcoma Center consented registry, based upon a diagnosis of metastatic/relapsed STS in patients aged 18 years or older who had received at least two lines of systemic therapies with initiation of second-line systemic therapy between January 1, 2000 and February 4, 2011. In addition, documentation of confirmed disease progression on or after first-line therapy and at least 3 months of data following commencement of a second-line therapy were also required.

Initially, data collection focused on patients with leiomyosarcoma; however, enrollment was subsequently expanded to include a broad range of STS histological subtypes. Patients were excluded if they had been diagnosed with GIST, bone sarcoma, or dermatofibrosarcoma protuberans, or had received treatment with pazopanib (including experimental use). Patient data were abstracted into an electronic case report form that was independently reviewed and queried. Unless otherwise specified, analyses presented here include all eligible patients and histological subtypes.

For the analysis, the variables collected from the medical records included patient demographics, histological subtype, treatment type and duration for all lines of systemic therapy, adverse events leading to treatment modifications (e.g. discontinuation), overall survival, clinician-reported tumor response rate, and progression-free survival (PFS; calculated as time to clinician-reported tumor progression or date of death, whichever came first). Clinician-reported responses were based on imaging results, if available, and/or clinical assessment notes by the treating physician. The treating oncologist’s written evaluation in the medical record was used to define a tumor response, rather than strictly limited to the use of formal oncology criteria such as Response Evaluation Criteria in Solid Tumors (RECIST) to define “objective responses.”

For the purpose of data analysis, patients were categorized according to the following types of second-line systemic therapy received: gemcitabine-based (including gemcitabine plus docetaxel), anthracycline-based (including anthracycline combined with other agents like ifosfamide), alkylating agents (including ifosfamide monotherapy), taxane-based, investigational agents (trabectedin, angiogenesis inhibitors), or other.

Continuous variables were summarized as means with standard deviation or median and range, as appropriate. Categorical variables were summarized by absolute frequencies and percentages. Time-to-event statistics were computed using Kaplan-Meier survival analyses.

## Results

Of the 99 patients with metastatic/relapsed STS included in this analysis, the mean age was 51.9 years and 62% were women (Table 1). Approximately one-half of the patients (48%) had leiomyosarcoma, and about 67% of the patients in this referral center population had participated in a clinical trial at some point in their care.

**Table 1:** **Patient demographics and clinical characteristics**

Patients evaluated	N = 99
Female, %	62
Median age, years	51.9
Patients alive at time of data abstraction, %	37
History of surgery, %	87
Histological subtype, %	
Leiomyosarcoma	48
Synovial cell sarcoma	7
Liposarcoma	5
Other:	39
*Alveolar soft part sarcoma*	5
*Clear cell sarcoma*	4
*Dedifferentiated liposarcoma*	4
*Solitary fibrous tumor*	4
*Endometrial stromal sarcoma*	4
*Adenosarcoma*	3
*Carcinosarcoma*	2
*Fibrous histiocytoma*	2
*PEComa*	2
*Epithelioid and round cell malignant neoplasm, desmoplastic small round-cell sarcoma, epithelioid sarcoma, epithelioid hemangioendothelioma, myxoid liposarcoma, rhabdomyosarcoma, round cell sarcoma, spindle cell sarcoma, undifferentiated sarcoma, and uterine sarcoma*	1 Each
Reason for discontinuing second-line therapy, %	
Progressive disease	73
Completed therapy course	10
Adverse events	6
Surgery	2
Death	1
Developed second primary malignancy^a^	1
Patient request	1
Avoidance of cumulative toxicity	1
Unclear	6

^a^One patient developed a new primary colon carcinoma.

The primary reason for treatment discontinuation of second-line therapy was progressive disease, which accounted for 73% of patients (Table 1). Only 10% were considered to have “completed” their course of therapy prior to developing progressive disease or discontinuing due to adverse events. Six percent of patients discontinued second-line therapy due to an adverse event.

**Figure 1:**
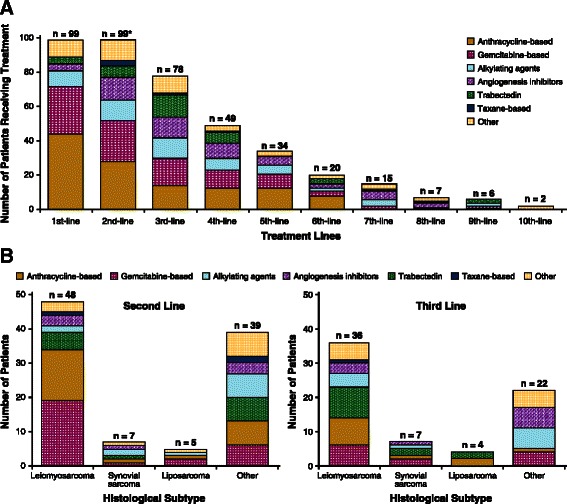
**Systemic therapy treatment patterns.** Treatment patterns according to **(A)** therapy line, and **(B)** second-line and third-line systemic therapy received according to histological subtype. *Patients were required to have at least one second-line systemic therapy for soft tissue sarcomas to be eligible for this study. Therefore, the first-line therapy distribution was based on those patients receiving at least one additional line of therapy (i.e. second-line or second-line plus additional lines of therapy).

One selection criterion for this study was that all patients were required to have had first- and second-line systemic therapies. This study population then received subsequent systemic therapy as follows: 78.8% had third-line, 49.5% had fourth-line, and 35.5% received fifth-line therapies. The maximum number of lines of systemic therapies received was 10. Figure 1A summarizes these treatments.

For patients receiving first-line treatment, 44% received anthracycline-based therapy, and a significant fraction (28%) received gemcitabine-based regimens as first-line therapy (Figure 1A). For patients with leiomyosarcoma, no clear difference was seen in the initial treatment regimens received by patients with uterine leiomyosarcoma versus non-uterine leiomyosarcoma (Table 2).

**Table 2:** **Comparison of first-line treatments for uterine versus non-uterine LMS**

Treatment	Uterine LMS	Non-uterine LMS	Total LMS
	(n = 24),	(n *= 2*4),	(N = 48),
	n (%)	n (%)	n (%)
Anthracycline-based	7 (29.2)	11 (45.8)	18 (37.5)
Gemcitabine-based	11 (45.8)	10 (41.7)	21 (43.8)
Alkylating agents	1 (4.2)	1 (4.2)	2 (4.2)
Angiogenesis inhibitors	1 (4.2)	1 (4.2)	2 (4.2)
Trabectedin	2 (8.3)	1 (4.2)	3 (6.3)
Other	2 (8.3)	0 (0.0)	2 (4.2)

*Abbreviation: LMS* leiomyosarcomas.

For patients receiving second-line systemic therapy, gemcitabine-based therapies were commonly used (28%), with a similar percentage of patients receiving anthracycline-based therapies (24%) (Figure 1A). Second- and third-line treatment patterns according to histological subtype did not suggest any clear trends (Figure 1B).

Median PFS from initiation of second-line treatment varied somewhat across regimens, ranging from 2.0 to 6.6 months (overall median [95% confidence interval (CI)], 5.4 months [3.3–7.0]). Median PFS (95% CI) was 6.4 months (3.0–16.5) for gemcitabine-based therapy (n = 28), 5.8 months (2.3–8.0) for anthracycline-based therapy (n = 24), 3.7 months (1.4–13.6) for trabectedin (n = 13), 2.0 months (0.7–5.1) for alkylating agents (n = 12), 6.5 months (1.0–8.4) for angiogenesis inhibitors (n = 7), 4.7 months (2.0–7.0) for taxane-based agents (n = 3), and 6.6 months (2.4–18.1) for other agents (n = 7).

Median duration for second-line treatment across all regimens was 4.1 months (95% CI, 3.0–5.0). A clinician-documented response was observed in less than 20% of patients during any individual line of systemic therapy (Table 3). Just over one-third of patients (n = 38/99 [38%]) had a clinician-documented response to any line of chemotherapy. The mean duration of any line of therapy beyond first-line treatment was in the range of 2 to 6 months (Table 3), documenting the frequent incidence of treatment discontinuation or switching, possibly due to lack of efficacy, toxicity, patient intolerance, or other adverse factors.

**Table 3:** **Treatment outcomes according to line of therapy**

Line of systemic therapy	Patients receiving therapy, n	Clinician-documented response, n (%)	Treatment duration, months, mean (SD)	Time from initiation of previous therapy to current therapy, months, mean (SD)
First-line	99	19 (18.8)	6.0 (7.4)	N/A
Second-line	99	15 (15.2)	5.4 (5.3)	12.3 (13.4)
Third-line	78	8 (11.1)	4.1 (4.2)	9.9 (12.1)
Fourth-line	49	5 (11.4)	5.3 (9.1)	6.2 (5.6)
Fifth-line	35	4 (12.5)	4.2 (5.2)	5.0 (3.6)
Sixth-line	20	2 (11.1)	3.2 (3.7)	7.3 (7.4)
Seventh-line	15	1 (7.7)	1.7 (1.1)	5.7 (6.0)
Eighth-line	7	1 (16.7)	4.1 (3.3)	5.7 (6.5)
Ninth-line	6	0 (0.0)	2.2 (2.2)	7.9 (9.6)
Tenth-line	2	0 (0.0)	3.9 (5.0)	4.4 (1.6)
Most recent therapy	99	13 (12.9)	3.6 (6.3)	8.7 (9.6)

*Abbreviations: N/A* not available; *SD* standard deviation.

**Figure 2:**
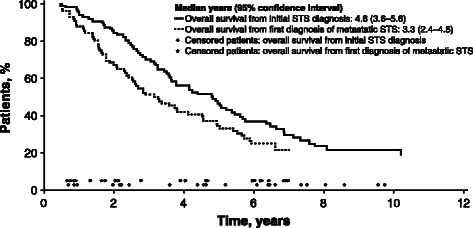
**Overall survival from initial diagnosis of STS and from diagnosis of metastatic/relapsed STS.***Abbreviation: STS* soft tissue sarcoma.

The median overall survival from initial STS diagnosis was 4.8 years (95% CI, 3.6–5.6) in this highly selected referral center population of patients with metastatic/relapsed STS (Figure 2). The median overall survival from first diagnosis of metastatic STS was 3.3 years (95% CI, 2.4–4.5).

## Discussion

The goals of this retrospective study were to assess treatment patterns for metastatic/relapsed STS and to identify whether any consistent “standard of care” for metastatic/relapsed STS could be identified in clinical practice from this population of patients generally treated in the community prior to referral to a tertiary care academic medical center in the United States. The only general trend identified was in the choice of initial treatment regimen. Most patients received anthracycline-based and gemcitabine-based therapy for first-line and second-line treatment, respectively. Notably, a high percentage of patients received gemcitabine-based therapy as first-line treatment of STS, and these patients generally received anthracycline-based therapy upon failure of the first-line regimen. However, no clear patterns for third-line therapy and beyond were noted. In addition, there was no clear link between histological subtypes and any particular treatment patterns.

A retrospective study describing international treatment patterns, the Sarcoma Treatment and Burden of Illness in North America and Europe (SABINE) study, similarly found that anthracycline-based regimens were most commonly used as first-line therapy in metastatic/relapsed STS [6]. The most common STS subtype in the SABINE study was leiomyosarcoma (46.5%). Doxorubicin monotherapy (34%) or an anthracycline (doxorubicin or epirubicin) combined with ifosfamide (30%) were identified as the most common first-line treatments in the SABINE study, which contrasts to more diverse first-line options in this study with 44% of patients receiving anthracycline-based therapy. The most common second-line treatment in the SABINE study was gemcitabine plus docetaxel (18.0%). This is similar to the 28% of patients who received gemcitabine-based therapy in the present study—who predominantly received gemcitabine plus docetaxel. The common use of gemcitabine in both studies could be related to the high proportion of patients with leiomyosarcoma in this study sample. The SABINE study found that trabectedin, which is approved for use in Europe but is only available as an investigational agent in the United States at specific centers, was commonly used after failure of first- and second-line therapy, which is consistent with results here. Similar to the observations noted in our study, the proportion of favorable responses to chemotherapy in the SABINE study declined with additional lines of treatment.

The majority of patients in the current study discontinued second-line treatment due to progressive disease and received additional lines of treatment. Less than 20% of these patients had a favorable response to treatment during any line of therapy (Table 3).

The median overall survival from diagnosis of metastatic disease was 3.3 years (39 months) in our study, which was considerably longer than the 10 to 18 months frequently reported in the literature for metastatic/relapsed STS [10,17,18]. However, the SABINE study reported a similar median overall survival from diagnosis of metastatic disease of 33.3 months [6]. It was not possible to assess the contribution of referral bias of more fit patients to tertiary care sarcoma centers versus the value of highly coordinated expert care delivered by sarcoma-dedicated teams, but these components could have contributed to longer survival. It is also likely that patients who go on to receive at least two rounds of chemotherapy have a better prognosis than those patients who might be too frail to receive chemotherapy. The high proportion of patients participating in clinical trials (67%) is also consistent with the hypothesis that this referral center patient population is likely to have fewer adverse clinical factors that could negatively influence survival.

Although data suggest that survival has somewhat improved in patients with sarcoma over the past two decades, the overall outcomes of metastatic STS remain poor. Italiano and colleagues [18] reported that overall survival of patients with metastatic/relapsed STS in the French Sarcoma Group database improved from 14 months (1987–1991) to 18 months (2002–2006). Multimodal treatment approaches may account for some improvement in survival over time [19,20]. Despite these improvements, a pressing need remains for more effective treatment options of these life-threatening diseases.

Effectiveness results from this study may not be fully representative of the broader metastatic/relapsed STS population given the selected patient population treated in a single tertiary sarcoma center. This retrospective analysis also used a practice-based approach to defining criteria for response rates and disease progression compared with the rigorously standardized criteria used in a clinical trial setting (e.g. RECIST), which may also confound direct comparisons between the effectiveness data from such a practice-based review and prospectively defined clinical research trials. In addition, the small number of patients included from a single tertiary care center led to wide CIs, particularly in the subtypes, and may limit the generalizability of these findings, although the data reviewed included community-based practice assessments of treatment regimens prior to referral to the academic center.

STS represents a diverse and varied collection of histological subtypes with distinct biological characteristics, natural histories, and responses to treatment. Because of the heterogeneity of patients receiving later lines of therapy, conclusions about these very limited subsets of the STS population should be made with care. Considering histological subtypes, the STS subtypes represented in our study do not reflect the natural distribution, and only leiomyosarcomas, which comprised 48% of our sample, were adequately represented. Of note, 39% of the population was derived from “other” subgroups, thus making assessment difficult and clouding the ability to identify uniform standards.

## Conclusions

This retrospective analysis from a large academic cancer center shows wide variation in treatment patterns, including switching between anthracycline- and gemcitabine-based therapy in early lines and significant heterogeneity in decisions regarding later lines of treatment. The majority of patients discontinued second-line treatment due to progressive disease and often received additional lines of treatment, with frequent switching of treatment. A significant unmet medical need exists for effective treatments among patients with metastatic/relapsed STS.

## Abbreviations

GIST: Gastrointestinal stromal tumor
PFS: Progression-free survival
RECIST: Response Evaluation Criteria in Solid Tumors
SABINE: Sarcoma Treatment and Burden of Illness in North America and Europe
STS: Soft tissue sarcoma
